# Large-scale real-world data analysis identifies comorbidity patterns in schizophrenia

**DOI:** 10.1038/s41398-022-01916-y

**Published:** 2022-04-11

**Authors:** Chenyue Lu, Di Jin, Nathan Palmer, Kathe Fox, Isaac S. Kohane, Jordan W. Smoller, Kun-Hsing Yu

**Affiliations:** 1 Department of Biomedical Informatics, Harvard Medical School, Boston, MA USA; 2 Computer Science and Artificial Intelligence Laboratory, Massachusetts Institute of Technology, Cambridge, MA USA; 3 Department of Psychiatry, Massachusetts General Hospital, Boston, MA USA; 4 Department of Epidemiology, Harvard T.H. Chan School of Public Health, Boston, MA USA

**Keywords:** Schizophrenia, Physiology

## Abstract

Schizophrenia affects >3.2 million people in the USA. However, its comorbidity patterns have not been systematically characterized in real-world populations. To address this gap, we conducted an observational study using a cohort of 86 million patients in a nationwide health insurance dataset. We identified participants with schizophrenia and those without schizophrenia matched by age, sex, and the first three digits of zip code. For each phenotype encoded in phecodes, we compared their prevalence in schizophrenia patients and the matched non-schizophrenic participants, and we performed subgroup analyses stratified by age and sex. Results show that anxiety, posttraumatic stress disorder, and substance abuse commonly occur in adolescents and young adults prior to schizophrenia diagnoses. Patients aged 60 and above are at higher risks of developing delirium, alcoholism, dementia, pelvic fracture, and osteomyelitis than their matched controls. Type 2 diabetes, sleep apnea, and eating disorders were more prevalent in women prior to schizophrenia diagnosis, whereas acute renal failure, rhabdomyolysis, and developmental delays were found at higher rates in men. Anxiety and obesity are more commonly seen in patients with schizoaffective disorders compared to patients with other types of schizophrenia. Leveraging a large-scale insurance claims dataset, this study identified less-known comorbidity patterns of schizophrenia and confirmed known ones. These comorbidity profiles can guide clinicians and researchers to take heed of early signs of co-occurring diseases.

## Introduction

Schizophrenia is a complex and chronic mental illness accompanied by major impairments in mental and social functioning [1]. It is one of the most disabling psychiatric disorders, affecting >3.2 million individuals in the USA [2] and costing ~$155.7 billion per year [3]. Core symptoms of schizophrenia are categorized as positive, negative, and cognitive symptoms [4]. Positive symptoms are the most easily identified and include hallucinations, delusions, and suspiciousness [5]. Negative symptoms are characterized by deficits of mental or emotional functioning such as impaired attention, avolition, alogia, and anhedonia [6]. Cognitive symptoms involve issues with concentration and memory such as impaired executive function and working memory, and disorganized speech or thoughts [7]. The course of schizophrenia is typically chronic, with only 20% of patients reporting favorable treatment outcomes and the remaining experiencing numerous psychotic episodes and persistent symptoms [5].

Real-world data, such as electronic health records (EHRs) and health insurance claims datasets, are playing an increasingly important role in informing healthcare decisions [8]. These data capture the actual interactions between patients and the healthcare system and provide insights into the clinical courses of individual patients at scale [9]. In the past decade, researchers have developed several population-level phenotype profiling methods based on EHR or health insurance claims data, which enable large-scale analyses of real-world healthcare data. One such effort is the compilation of the phenome-wide association study codes (phecodes), which maps the International Statistical Classification of Diseases and Related Health Problems (ICD) codes to distinct phenotype categories [10, 11]. This effort enables evaluating the associations among a large number of phenotypes and genetic variations [12–14]. Insurance claims datasets provide a rich and unique data source for phenotypical analyses: these datasets contain longitudinal health histories, including drug prescriptions, laboratory tests, procedure codes, and patients’ diagnoses in ICD codes [15], and they comprise millions of patients, enabling large-scale phenome-wide analyses. Population-level phenotypic analyses using phecodes based on insurance claims data have successfully revealed the progression of disease phenotypes in many previous studies [16–22].

A few prior studies examined the common comorbidities of schizophrenia patients [23]. However, these analyses are typically based on small-scale observational studies and clinical trials with up to hundreds of participants [24, 25], and are typically limited in the demographic and geographic diversity of patient cohorts [26]. To address this gap, we leveraged a nationwide health insurance claims dataset from Aetna, which encompassed >86 million participants in the USA, to conduct a population-level study of phenotypes associated with schizophrenia. We conducted data-driven analyses on the patients’ phenotypes before and after the index diagnosis of schizophrenia and compared phenotypic profiles between schizophrenic patients and matched non-schizophrenic participants. In addition, we systematically compared the phenotypic differences between schizoaffective disorder and other types of schizophrenia. Our systematic analyses enabled the discovery of previously under-reported risk factors and comorbidities of schizophrenia at a population scale.

## Methods

### Study participants and dataset summary

Using un-identifiable member claims data from Aetna, we identified the International Statistical Classification of Diseases and Related Health Problems (ICD) versions 9 and 10 code profiles of each member. The Aetna dataset contains 86 million participants in total with claims data from January 2008 to December 2019. At the time of participants’ enrollment in their insurance plans, written informed consent was obtained. This employer-sponsored health insurance includes participants representing all 50 states, the District of Columbia, five populated territories, and Armed Forces Europe, with the most patients from Texas, California, Pennsylvania, Florida, and New York. The dataset records a multitude of patients’ healthcare-related information, including ICD diagnostic codes, procedures in Current Procedural Terminology (CPT) codes, prescribed drugs in National Drug Codes (NDC), laboratory test results, and basic demographics, such as age and sex. To reduce dimensionality, we mapped each ICD code to its corresponding phecode. Phecodes used in this study are based on both ICD-9 and ICD-10 codes. For example, the phecode 1000 “Burns” contains 388 ICD-9 codes and 3,708 ICD-10 codes related to burns, such as ICD-9 code 940.0 “chemical burn of eyelids and periocular area” and ICD-10 code T23.16 “burn of first degree of back of hand”.

We define schizophrenia patients as participants with at least three occurrences of phecodes 295 and 295.1. Three occurrences were required in determining the presence of schizophrenia to minimize the impact of clinical miscoding, and studies using insurance claims datasets commonly use the same three-hit threshold [27–30]. Patients were included if they had documented birth year, biological sex, and zip code information and had been in the insurance plan for at least 12 months prior to their first diagnosis of schizophrenia. We excluded patients <15 years of age because early-onset schizophrenia is rare and its clinical presentations overlap with developmental and mood disorders, leading to a relatively high level of miscoding in this age group [31]. Each patient in the schizophrenia cohort was matched to a non-schizophrenic control participant of the same birth year, biological sex, and the first three digits of their zip codes. Control participants had no mention of phecodes 295 and 295.1 during their enrollment periods and were enrolled in the claims database at least 1 year prior to their matched patient’s schizophrenia first diagnosis date in the database (the index date). These stringent criteria decrease the uncertainty of clinical conditions extracted from the dataset. Due to the short observation period, we cannot reliably capture the true dates of schizophrenia onset in older adults. Thus, the index date for older participants may represent the date at which schizophrenia resurfaced as an active clinical issue. Harvard Medical School Institutional Review Board approved this study (IRB18-0198).

### Systematic analyses of the prevalence of preceding phenotypes of schizophrenia

We conducted comprehensive analyses on each of the 1890 phenotypes defined by the phecodes and reported significantly enriched or depleted phenotypes before the diagnosis of schizophrenia. We focused this analysis on patients whose first schizophrenia documentation occurred between the ages of 15 and 29, because schizophrenia onset usually occurs in the late teens and twenties [32], and given the relatively short observation period of insurance claims datasets, late-onset schizophrenia cases are challenging to ascertain. In identifying the preceding phenotypes, we examined the prevalence of each phecode in the schizophrenic group and the matched control group. To enhance specificity and reduce the impact of coding errors, a phecode was deemed as present only when at least three ICD codes were documented for a given patient. We filtered out phenotypes with <10 patients in either the schizophrenic or the non-schizophrenic group. Fisher’s exact test was performed to evaluate prevalence differences of a phecode of interest between the schizophrenic group and the matched control group. Consistent with standard large-scale systematic studies, we employed one of the more stringent corrections, Bonferroni adjustment, to correct for multiple comparisons of the remaining phenotypes after the filtering steps. We reported the phenotypes predictive of schizophrenia as those with a corrected *p*-value < 0.05 and ranked them by their odds ratios. We visualized the results with a Manhattan plot using the *PheWAS* R package, ordered by broader disease categories [33], as well as a volcano plot. Sex-stratified analyses were conducted to identify the phenotypical differences in schizophrenic men and women. To ensure the robustness of the analyses, we filtered out phenotypes with <5 participants in either schizophrenia or non-schizophrenia group. This lower filter threshold of five participants is to account for the smaller sample sizes in the sex-stratified analyses.

### Systematic disease risk comparison between schizophrenic and non-schizophrenic groups

To identify the phenotypes occurring after the onset of schizophrenia, we again required a minimum of three codes within a given phecode after the schizophrenia index date. For each phecode analysis, we removed patients who had the phecode within 1 year (the wash-out period) prior to the index date. For patients who did not have the phecode after schizophrenia, their last dates in the database were used as their censored dates. For each phecode, we used univariate Cox proportional hazard models and obtained the hazard ratios and *p*-values to evaluate the difference between the two patient groups. Phecodes with <10 patients in either the schizophrenic or matched control groups were removed. We reported phecodes with Bonferroni-adjusted *p*-values of <0.05. Sex-stratification analyses were conducted similarly and phenotypes with <5 participants were removed. Additionally, the same analysis was repeated for three different age groups: 15–29, 30–59, as well as 60 and above.

### Systematic Disease Risk Comparison between Schizophrenia and Schizoaffective Disorders

The phecodes for schizophrenia capture both schizophrenia and schizoaffective disorder. However, schizoaffective disorder has its own ICD code and diagnostic criteria in the fourth and fifth editions of the Diagnostic and Statistical Manual of Mental Disorders (DSM-IV and DSM-V). To evaluate the phenotypic differences between patients with schizoaffective disorder and those with schizophrenia, we identified ICD codes of schizoaffective disorder and those of schizophrenia without the affective component, and we separated the schizophrenic cohort into patients with schizoaffective disorders and patients with other types of schizophrenia. We removed patients with mentions of both groups and compared the succeeding phecodes of patients in these two groups, because it is difficult to verify their actual diagnoses in this dataset. Phecodes with <5 patients in either the schizoaffective or other schizophrenia groups were removed to mitigate effects from outliers. We reported phecodes with Bonferroni-adjusted *p*-values < 0.05.

## Results

### Patients characteristics

We identified 61,453 patients with schizophrenia from the insurance claims dataset. Approximately half of the patients (47.2%) were male, consistent with the previously reported population distribution of schizophrenia patients [1]. The average length of patient records in our study cohort is ~6 years. Patients in the cohort represent all 50 states, the District of Columbia, five permanently inhabited territories, and Armed Forces Europe. Supplemental Fig. 1 shows the study flowchart. Details of patient characteristics can be found in Supplemental Table 1 and Supplemental Figs. 2 and 3.

### Phenotypes preceding the diagnosis of schizophrenia

**Fig. 1:**
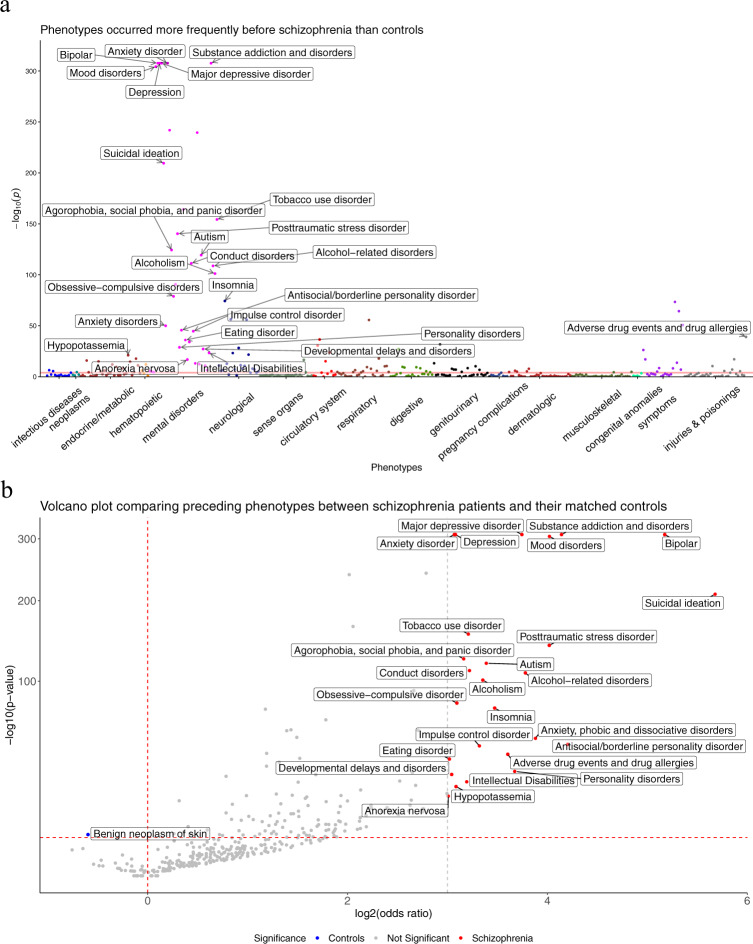
Systematic analysis of phenotypes preceding a diagnosis of schizophrenia. **a** Manhattan plot of the phenotypes, grouped by disease categories. The red horizontal line indicates a Bonferroni-corrected *P*-value threshold. Phenotypes with odds ratios (OR) > 8 are annotated. **b** Volcano plot of *P*-value (in −log_10_) versus the OR (in log_2_). The red horizontal dashed line indicates the same Bonferroni-corrected *P*-value threshold, the red vertical dashed line designates OR = 1, and the gray vertical dashed line indicates an OR = 8 threshold, above which phenotypes enriched in the schizophrenic group are annotated.

To identify the phenotypes preceding a diagnosis of schizophrenia, we identified each of the 1890 phenotypes (*P*) encoded in the phecodes and counted the number of patients with *P* in both the schizophrenia patient cohort and the matched control group. Results showed that 144 phecodes are significantly enriched (Bonferroni-corrected *P*-value < 0.05) in patients who later received schizophrenia diagnoses (Fig. 1), with one phecode enriched in controls. The top ten phenotypes, sorted by odds ratios, enriched in schizophrenic patients are presented in Table 1. Further stratification by sex revealed that women who later developed schizophrenia are more likely to have type 2 diabetes, obstructive sleep apnea, and eating disorders, while men who later developed schizophrenia are more likely to have conditions such as acute renal failure, developmental delays, and rhabdomyolysis (Supplemental Table 2 and 3).

**Table 1:** Top ten significantly enriched phenotypes preceding schizophrenia diagnoses in adolescents and young adults aged between 15 and 29 (number of schizophrenia patients in this age group = 13,828).

Phecode	Phecode description	Number of schizophrenia patients	Number of non-schizophrenic participants	Odds ratio	95% Confidence interval	*P*-value
297.1	Suicidal ideation	775	16	51.2	(31.3, 90.2)	3 × 10^−210^
296.1	Bipolar	2476	83	36.1	(29, 45.5)	2 × 10^−308^
301.2	Antisocial/borderline personality disorder	200	11	18.4	(10.1, 37.5)	2 × 10^−46^
316	Substance addiction and disorders	2635	182	17.6	(15.1, 20.7)	2 × 10^−308^
296	Mood disorders	1369	93	16.2	(13.1, 20.3)	5 × 10^−305^
300.9	Posttraumatic stress disorder	636	41	16.2	(11.8, 22.8)	5 × 10^−141^
300	Anxiety, phobic and dissociative disorders	232	16	14.7	(8.9, 26.2)	1 × 10^−50^
317	Alcohol-related disorders	517	39	13.7	(9.9, 19.6)	2 × 10^−109^
296.22	Major depressive disorder	3406	329	13.4	(11.9, 15.1)	2 × 10^−308^
301	Personality disorders	139	11	12.8	(6.9, 26.1)	2 × 10^−29^

### Data-driven analysis revealed the comorbidities after the onset of schizophrenia

**Fig. 2:**
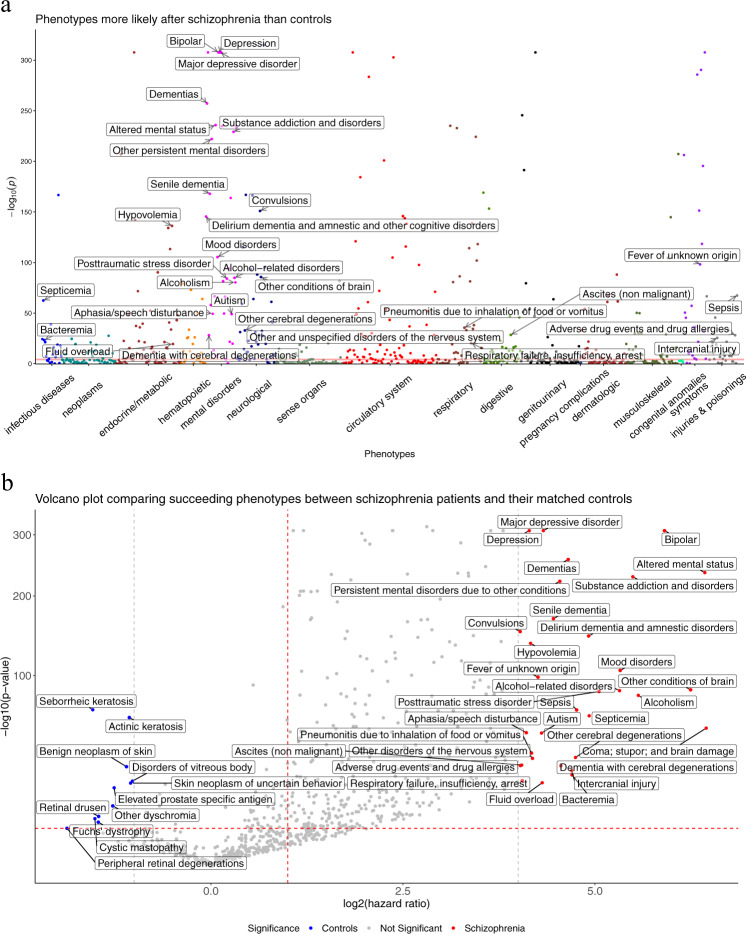
Systematic analysis of the phenotypes following the clinical diagnosis of schizophrenia. **a** Manhattan plot of the phenotypes. The red horizontal line indicates a Bonferroni-corrected *P*-value threshold. Annotated phenotypes have hazard ratios (HRs) ≥16. **b** Volcano plot of the phenotypes. The red horizontal dashed line indicates the same Bonferroni-corrected *P*-value threshold, the red vertical dashed line designates an HR = 1 threshold, and the gray vertical dashed lines indicate HR = 16 and HR = 0.5. Phenotypes with more extreme HRs are labeled in the figure.

Using a Cox proportional hazard model, we found 402 phecodes are significantly enriched (Bonferroni-corrected *P*-value < 0.05) in the schizophrenic group following the disease onset (Fig. 2), with 24 phecodes significantly enriched in controls after their index dates. The results are summarized in the Manhattan plot and volcano plot in Fig. 2a, b. Table 2 shows the top ten phenotypes with the highest hazard ratios after the first diagnosis of schizophrenia in the dataset. We tested Cox proportional hazard assumptions using the survminer R package, and only one (316: Substance addiction and disorders) of the top ten phecodes failed the proportionality assumption after Bonferroni correction of test *p*-values. Sex-stratification analysis shows that women with schizophrenia are more likely to develop encephalopathy, epilepsy, and adverse drug events and allergies, while men with schizophrenia are more likely to develop impulse control disorder, esophageal bleeding, and acute osteomyelitis (Supplemental Tables 4 and 5).

**Table 2:** Top ten significantly enriched phenotypes after schizophrenia diagnoses overall (total number of schizophrenia patients = 61,453).

Phecode	Phecode description	Number of schizophrenia patients	Number of non-schizophrenic participants	Hazard ratio	95% Confidence interval	*P*-value
348.1	Coma; stupor; and brain damage	1017	12	87.3	(49.4, 154.2)	2 × 10^−53^
292.4	Altered mental status	4334	55	86.2	(66.1, 112.4)	2 × 10^−236^
348	Other conditions of brain	1547	21	75.8	(49.3, 116.6)	2 × 10^−86^
296.1	Bipolar	5990	108	60	(49.5, 72.5)	2 × 10^−308^
317.1	Alcoholism	1151	25	47.3	(31.8, 70.3)	4 × 10^−81^
316	Substance addiction and disorders	3181	74	45	(35.7, 56.7)	7 × 10^−230^
296	Mood disorders	1400	36	40.2	(28.8, 55.9)	4 × 10^−106^
317	Alcohol-related disorders	1133	29	40	(27.6, 57.7)	1 × 10^−85^
300.9	Posttraumatic stress disorder	1043	32	33.2	(23.3, 47.2)	9 × 10^−85^
038	Septicemia	731	25	30.4	(20.4, 45.2)	3 × 10^−63^

We further investigated disease risks stratified by three age groups: 15–29 years old (adolescents and young adults; 13,828 patients), 30–59 years old (“middle-aged” adults; 16,339 patients), and 60 years old and above (older adults; 31,286 patients). We found that substance addiction, anxiety, convulsions, and pain in the limb and neck are frequently reported in schizophrenia patients under 29 years old. Those between 30 and 59 years old tend to develop alcohol-related disorders, posttraumatic stress disorder, gait abnormality, syncope, and chronic kidney disease. Patients over 60 years old suffer from delirium, alcoholism, dementia, sepsis, fracture of the pelvis, osteomyelitis, and hypotension more often than their matched non-schizophrenic participants. (Supplemental Tables 6–8).

### Subtypes analysis identified distinct phenotypical patterns between schizophrenia and schizoaffective disorders

**Fig. 3:**
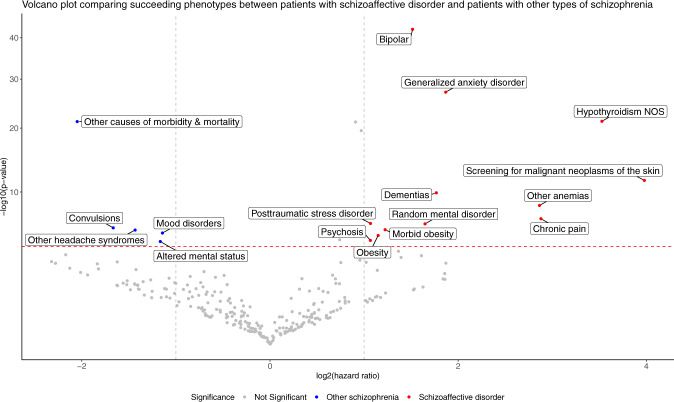
Phenotypical differences between schizoaffective disorder and other types of schizophrenia. Log_2_(HR) > 0 indicates phenotypes more frequently observed after the diagnoses of schizoaffective disorder, whereas log_2_(HR) < 0 indicates phenotypes more commonly observed after the diagnoses of other types of schizophrenia. The red horizontal dashed line indicates the Bonferroni-corrected *P*-value threshold. The gray vertical dashed lines indicate the HR of 2 and 0.5, respectively.

Although the standard phecodes for schizophrenia include both schizophrenia and schizoaffective disorder, schizoaffective disorder has distinct diagnostic criteria including a requirement for mood (manic or depressive) episodes. To systematically investigate the phenotypic difference between schizophrenia and schizoaffective disorder after their disease onset, we similarly conducted a data-driven phecode comparison. Of all patients with schizophrenia diagnoses, we obtained 3096 (4.91%) patients who had only schizoaffective disorder and 46,890 (74.4%) who had only schizophrenia or its subtypes. After matching each schizoaffective disorder patient with another patient with other types of schizophrenia of the same age and sex, we compared the succeeding phenotypes in these two groups. After the removal of phecodes with <5 participants in either group, 259 phecodes remained. Fig. 3 and Supplemental Tables 9–10 summarize the differences in succeeding high-risk phenotypes between schizoaffective disorder and schizophrenia.

## Discussion

We conducted a large-scale systematic analysis of the comorbidities of schizophrenia using a nationwide health insurance database. Consistent with the literature [34–39], we demonstrated that conditions such as anxiety, posttraumatic stress disorder, as well as alcohol and substance abuse are significantly more common in schizophrenic patients prior to their schizophrenia diagnoses. Benign neoplasm of skin was noted to be more prevalent in non-schizophrenic control patients, suggesting a higher frequency of routine health checkups. Of the significantly enriched phenotypes compared with their matched non-schizophrenic controls, women who later developed schizophrenia had a higher prevalence of type 2 diabetes, obstructive sleep apnea, and eating disorders than men. On the contrary, men had a higher prevalence of acute renal failure, rhabdomyolysis, and developmental delays. These results demonstrated the power of large-scale real-world data analyses in elucidating disease comorbidities systematically.

Our systematic analyses of phenotypes after the diagnosis of schizophrenia showed patients are at increased risk for experiencing many related psychiatric disorders, as noted above. In addition, these patients are at higher risk for conditions noted in previous literature, such as senile dementia [40] and epilepsy [41]. Additionally, the risk of eating disorders is enriched after schizophrenia diagnoses, which could stem from either their shared etiology [42] or antipsychotic medications for schizophrenia [43]. Findings from this study could inform clinicians in looking for early signs of these common comorbidities in schizophrenic patients and guide researchers to study the underlying etiology behind the identified comorbidity profiles. We found that men and women with schizophrenia have different comorbidity patterns. For example, women affected by schizophrenia are more likely to develop encephalopathy and adverse drug events, while men with schizophrenia had a higher risk of developing acute osteomyelitis. Our age-stratified analyses showed that, in addition to phenotypes discussed above, adolescents and young adults are likely to develop conduct disorders, insomnia, and antisocial and borderline personality disorder after schizophrenia diagnosis compared with non-schizophrenic controls. Middle-aged adults are more likely to develop phenotypes related to respiratory and renal failures, sepsis, convulsions, and pneumonia. Older adults are more likely to have phenotypes related to dementia, delirium, and sepsis. These results indicated that the evolving comorbidity patterns of schizophrenia across age groups. On the other hand, non-schizophrenic controls were more likely to have ICD codes related to routine health checkups on less debilitating symptoms, such as benign neoplasm of skin, elevated prostate-specific antigen, and keratosis. These findings suggest non-schizophrenia participants have better overall health and a higher tendency for seeking preventive care compared with those with schizophrenia. Previous work has developed schizophrenia prediction models [44, 45], which identified genetic and psychosocial risk factors of schizophrenia in smaller cohorts. Our study complements previous works by systematically examining the comorbidity landscape of schizophrenia and characterizes the full spectrum of associations between schizophrenia and other clinical phenotypes noted in the health insurance claims dataset in an unbiased analytical framework.

The use of ICD code groups also enabled us to investigate the more nuanced diagnoses within the broader category of schizophrenia. By comparing the risk of developing succeeding diseases between patients with schizoaffective disorder and those with other types of schizophrenia, we found significant differences in their comorbidity landscapes. Patients with schizoaffective disorders were at higher risk of developing anxiety disorders, dementia, and obesity. Although hypothyroidism NOS was enriched in patients with schizoaffective disorders, we did not observe such correlation when considering other phecodes related to hypothyroidism. It should also be noted that thyroid dysfunctions can sometimes be mistaken for mental disorders due to their similar clinical presentations [46]. Conversely, patients with other types of schizophrenia have higher risks for signs such as convulsions and altered mental status. Interestingly, mood disorders were more likely following this patient cohort, contradictory to the diagnostic criteria of schizoaffective disorder [47]. We hypothesize that clinicians in this dataset were less likely to document ICD codes related to mood disorders in patients already diagnosed and treated with schizoaffective disorder. It should be noted that there is a substantial level of clinical uncertainty in diagnosing schizoaffective disorders and schizophrenia, as the symptoms fall on a spectrum. Our data-driven analysis revealed the prevalence of clinically determined phenotypes that more frequently accompany one diagnosis than the other, which provided insights into the comorbidity clustering patterns under the current clinical diagnostic criteria and practice.

There are a few limitations in our real-world data analyses. First, this employer-sponsored insurance claims dataset from Aetna only includes patients and their family members and may not represent the general population. Some individuals with schizophrenia are covered by other private insurance, Medicaid, Medicare, or the Veterans Health Administration [48], and thus not included in our dataset. This inherent limitation of our dataset, combined with the fact that we used a conservative definition of schizophrenia (three records or more), was likely responsible for a lower schizophrenia prevalence than the estimate in the general population (0.7%). Second, the follow-up time is relatively short due to the nature of healthcare insurance datasets. Incomplete medical history limited our ability to discover very early signs of schizophrenia. To control for this, we conducted the preceding phenotype analyses only on late teens and young adults (15–29 years of age). In the succeeding phenotype analyses, we required a minimum of 1 year of observation before the index date, during which an eligible patient cannot have an associated schizophrenia code. These enrollment criteria increased the likelihood our index date is the onset date or the day schizophrenia resurfaced as a clinical issue. Third, ICD documentation in claims datasets can be uncertain, noisy, and subject to change over time [49]. For example, despite some overlap in symptoms, bipolar disorder and schizophrenia are mutually exclusive diagnoses based on DSM-V diagnostic criteria. Interestingly, we observed some patients who were initially diagnosed with bipolar disorder later received ICD-codes related to schizophrenia. Prior studies showed that 14.7% of patients receiving bipolar disorder as their initial diagnoses were identified as schizophrenia patients 10 years later [49]. These observations suggest the uncertainties in the initial diagnoses and the noisiness of the diagnostic codes extracted from EHRs. Therefore, cautious interpretation of results from EHR-driven analyses is necessary, and prior knowledge on the disease nosology may distinguish true comorbidities from spurious associations arising from mislabels. To quantitatively mitigate the uncertainty and noisiness of ICD coding, we applied a more stringent threshold for our inclusion criteria, requiring a minimum of three documented diagnoses. Our use of Bonferroni correction further decreases false-positive findings; lending improved reliability to our data-driven analyses. Lastly, although phecodes reduce the noise from inconsistent coding in different hospital systems and curtail its impact on our analyses, they only represent coarse categories of clinical phenotypes. For example, specific phenotypes within eating disorders (phecode: 305.2) have distinct etiologies and clinical presentations. Conditions such as bulimia nervosa, psychogenic loss of appetite, and cyclical vomiting are all categorized as eating disorders, obscuring more detailed clinical information. Future studies of EHR data examining individual ICD codes and incorporating natural language processing of the unstructured clinical notes could facilitate the characterization of more granular phenotypes.

Future research can further validate our findings in other large survey or registry databases [50, 51]. As an illustration, our systematic analyses revealed that obstructive sleep apnea is an enriched phenotype preceding the schizophrenia diagnoses in female patients. We hypothesize this finding is related to the connections among overweight, schizophrenia, and psychotropic medication [52]. These identified comorbidity patterns from our systematic analyses can help inform individualized predictive models for the onset of schizophrenia and its comorbidities [53]. Future studies can identify the causal genetic pathways, environmental mediators, or drug effects underpinning the observed associations. For example, studies have observed shared genetic risk between schizophrenia and immune-related disorders [54, 55]. As such, the identified correlations between schizophrenia and infections in this dataset may be partially explained by genetic predispositions.

In summary, our real-world data analyses revealed the common preceding and succeeding phenotypes of schizophrenia. We showed that psychiatric disorders, such as posttraumatic stress disorder and anxiety often precede and succeed the onset or new diagnosis of schizophrenia. Additionally, conduct disorders, eating disorders, and insomnia commonly followed schizophrenia onset among adolescents and young adults, and dementia and osteomyelitis occurred after older adults had a new diagnosis of schizophrenia. Such information could guide clinicians and researchers to look out for early signals and co-occurring diseases of schizophrenia in different age and sex groups, in order to help their patients better manage these illnesses.

## Supplementary information

Supplemental Materials

## Data Availability

The codes for conducting the analyses and the raw result tables could be found on a public GitHub repository: https://github.com/hms-dbmi/schizophrenia_comorbidity.
