# Supplementary material for: Large-scale real-world data analysis identifies comorbidity patterns in schizophrenia
Source: Transl Psychiatry. 2022 Apr 11;12:154. doi: 10.1038/s41398-022-01916-y (PMC9001711; doi:10.1038/s41398-022-01916-y)
Supplement: Supplementary file 1 — Supplemental Materials [file 41398_2022_1916_MOESM1_ESM.docx]

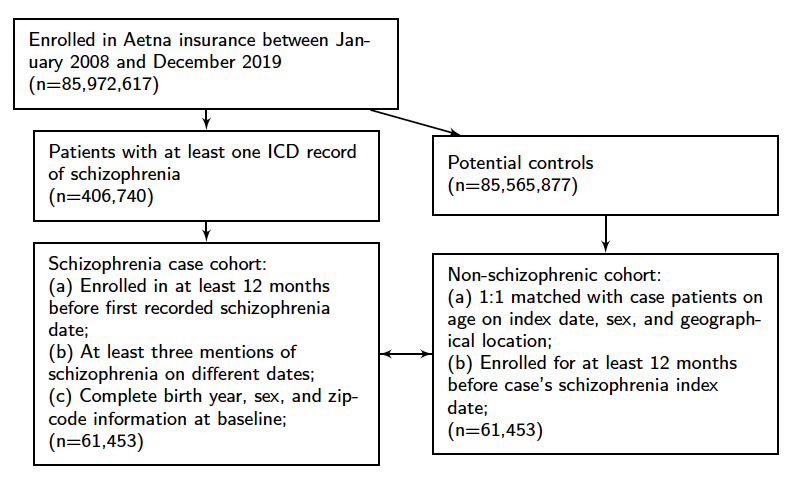


**Supplemental Figure 1.** Study flowchart.

**
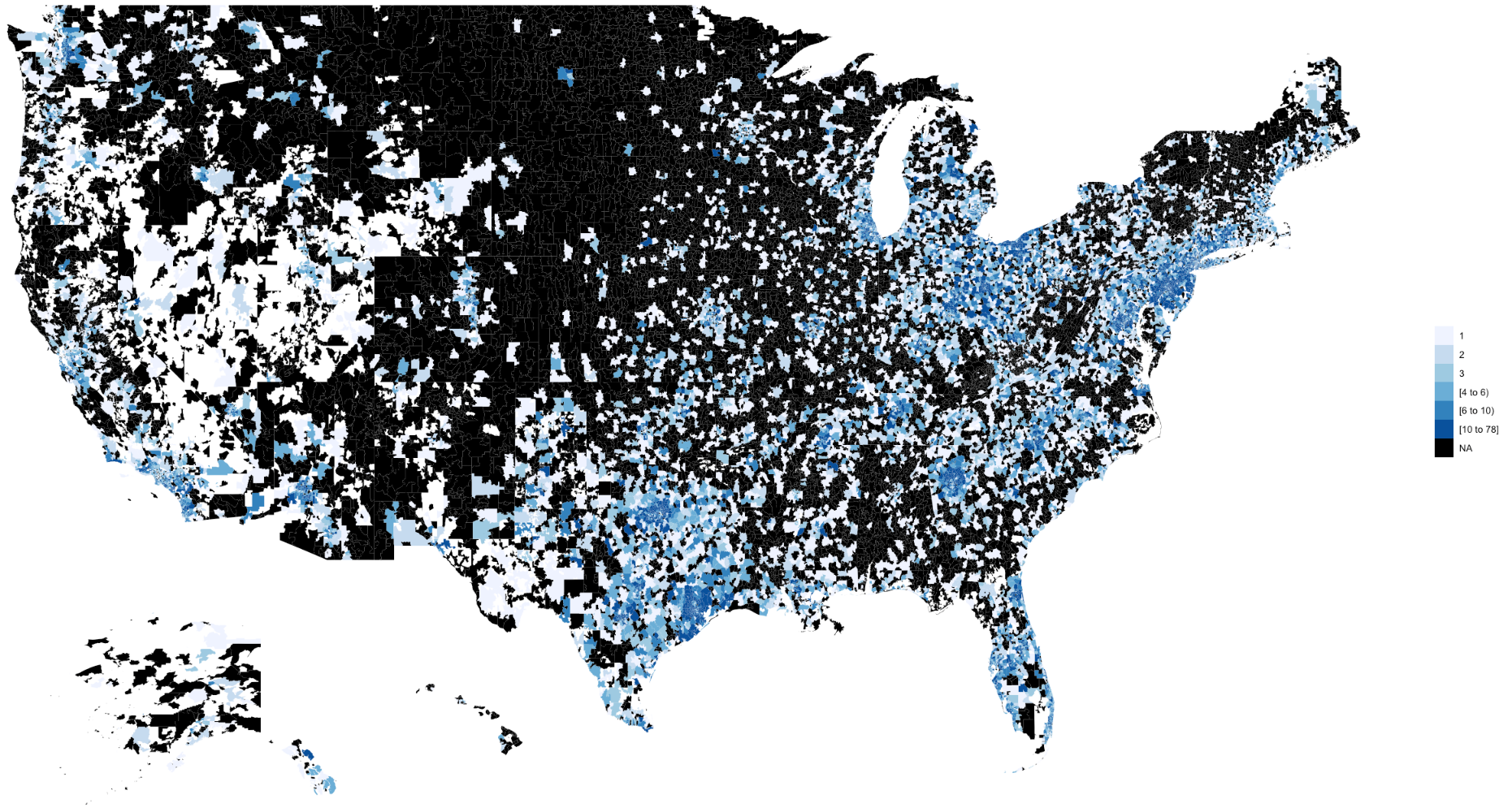
**

**Supplemental Figure 2.** Geographical distribution of patients included in this study.

**
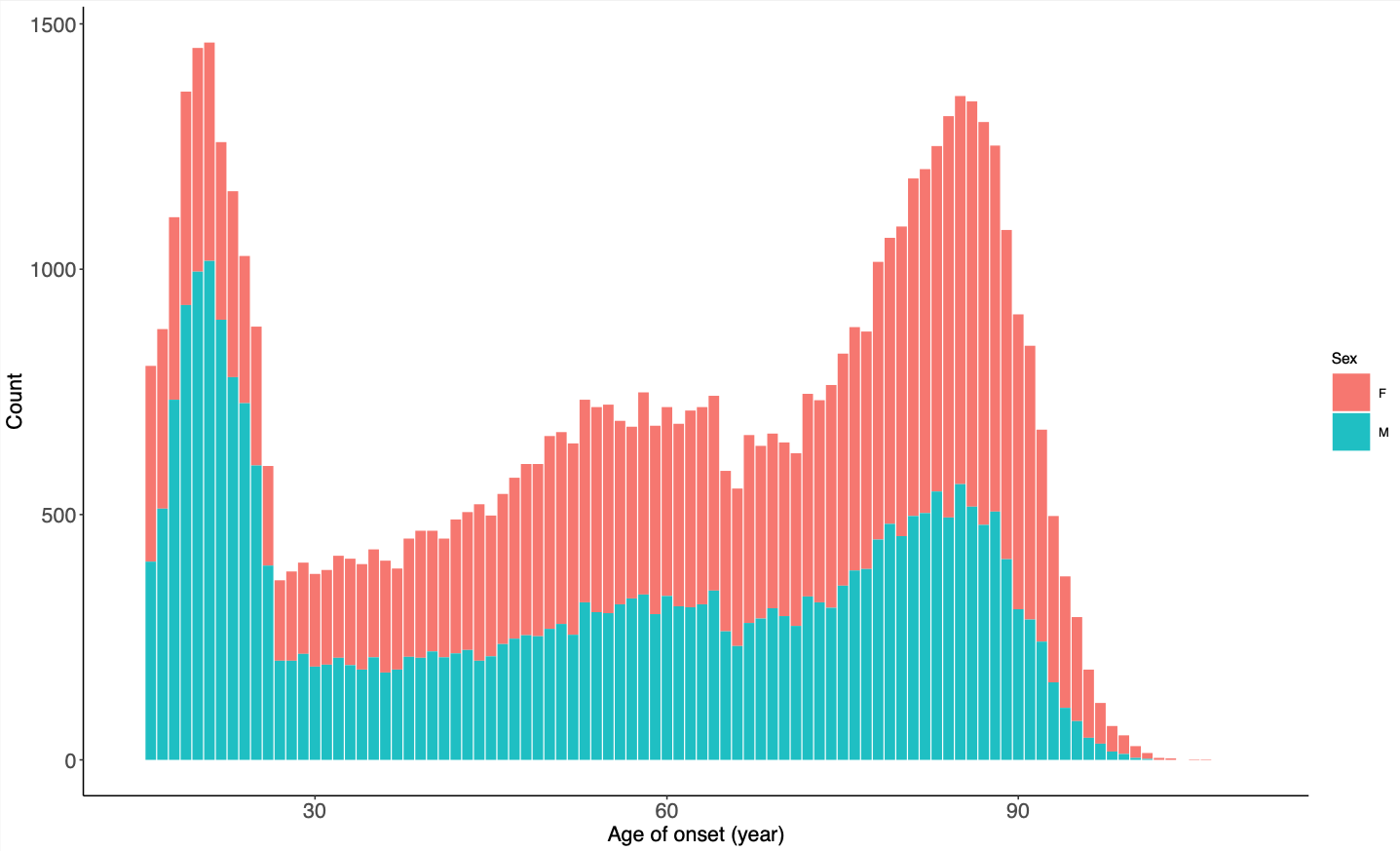
**

**Supplemental Figure 3.** Distribution of age at schizophrenia index date by sex.

**Supplemental Table 1.** Patient characteristics of the study cohort.

| Characteristics | Value (%) |
| --- | --- |
| Total patients | 61,453 |
| Males | 29,001 (47.2) |
| Females | 32,452 (52.8) |
| Days Enrolled |  |
| Average | 2177.6 |
| Minimum | 392 |
| Maximum | 4,382 |
| Age (in years) |  |
| Mean | 57.2 |
| Median | 60 |
| Geographic Region |  |
| East North Central | 10,232 (16.7) |
| East South Central | 11,008 (17.9) |
| Mid Atlantic | 12,334 (20.1) |
| Mountain | 2,882 (4.7) |
| New England | 2,885 (4.7) |
| Pacific | 5,405 (8.8) |
| South Atlantic | 12,418 (20.2) |
| West North Central | 2,057 (3.4) |
| West South Central | 11,008 (17.9) |

**Supplemental Table 2.** Top 10 phenotypes preceding schizophrenia in women but not in men between ages 15 and 29 (number of schizophrenia patients in this age group = 13,828).

| **Phecode** | **Phecode Description** | **Number of Female Schizophrenia Patients** | **Number of Female Non-Schizophrenic Participants** | **Odds Ratio** | **95% Confidence Interval** | **P-value** |
| --- | --- | --- | --- | --- | --- | --- |
| 301.2 | Antisocial/borderline personality disorder | 156 | 10 | 16.1 | (8.5, 34.2) | $3\times{10}^{-35}$ |
| 297.2 | Suicide or self-inflicted injury | 61 | 5 | 12.3 | (5.0, 39.4) | $2\times{10}^{-13}$ |
| 250.2 | Type 2 diabetes | 85 | 7 | 12.3 | (5.7, 31.6) | $3\times{10}^{-18}$ |
| 327.32 | Obstructive sleep apnea | 43 | 5 | 8.7 | (3.4, 28.0) | $1\times{10}^{-8}$ |
| 790.6 | Other abnormal blood chemistry | 40 | 5 | 8.1 | (3.2, 26.2) | $7\times{10}^{-8}$ |
| 305.2 | Eating disorder | 185 | 24 | 8.0 | (5.2, 12.8) | $1\times{10}^{-32}$ |
| 305.21 | Anorexia nervosa | 84 | 12 | 7.1 | (3.9, 14.3) | $1\times{10}^{-14}$ |
| 599.4 | Urinary incontinence | 40 | 6 | 6.7 | (2.8, 19.4) | $3\times{10}^{-7}$ |
| 350.1 | Abnormal involuntary movements | 36 | 6 | 6.0 | (2.5, 17.5) | $3\times{10}^{-6}$ |
| 327.3 | Sleep apnea | 27 | 5 | 5.4 | (2.1, 18.1) | $1\times{10}^{-4}$ |

**Supplemental Table 3.** Top 10 phenotypes preceding schizophrenia in men but not in women between ages 15 and 29 (number of schizophrenia patients in this age group = 13,828).

| **Phecode** | **Phecode Description** | **Number of Male Schizophrenia Patients** | **Number of Male Non-Schizophrenic Participants** | **Odds Ratio** | **95% Confidence Interval** | **P-value** |
| --- | --- | --- | --- | --- | --- | --- |
| 301 | Personality disorders | 75 | 7 | 10.8 | (5.0, 27.8) | $2\times{10}^{-15}$ |
| 1005 | Other symptoms | 38 | 5 | 7.6 | (3.0, 24.8) | $2\times{10}^{-7}$ |
| 315.1 | Learning disorder | 43 | 7 | 6.2 | (2.8, 16.3) | $2\times{10}^{-7}$ |
| 585.1 | Acute renal failure | 61 | 10 | 6.1 | (3.1, 13.4) | $4\times{10}^{-10}$ |
| 772.4 | Rhabdomyolysis | 40 | 7 | 5.7 | (2.5, 15.2) | $1\times{10}^{-6}$ |
| 315.3 | Intellectual disabilities | 74 | 13 | 5.7 | (3.2, 11.3) | $1\times{10}^{-11}$ |
| 315 | Developmental delays and disorders | 94 | 17 | 5.5 | (3.3, 10.0) | $4\times{10}^{-14}$ |
| 1008 | Internal injury to organs | 60 | 11 | 5.5 | (2.9, 11.6) | $2\times{10}^{-9}$ |
| 509.1 | Respiratory failure | 63 | 12 | 5.3 | (2.8, 10.8) | $2\times{10}^{-9}$ |
| 313.2 | Tics and stuttering | 74 | 16 | 4.7 | (2.7, 8.6) | $4\times{10}^{-10}$ |

**Supplemental Table 4.** Top 10 phenotypes succeeding schizophrenia in women but not in men (total number of schizophrenia patients = 61,453).

| **Phecode** | **Phecode Description** | **Number of Female Schizophrenia Patients** | **Number of Female Non-Schizophrenic Participants** | **Hazard Ratio** | **95% Confidence Interval** | **P-value** |
| --- | --- | --- | --- | --- | --- | --- |
| 348.1 | Coma; stupor; and brain damage | 534 | 8 | 68.5 | (34.1, 137.8) | $2\times{10}^{-32}$ |
| 290.2 | Delirium due to conditions classified elsewhere | 279 | 5 | 57.4 | (26.7, 138.9) | $3\times{10}^{-19}$ |
| 305.2 | Eating disorder | 170 | 8 | 21.4 | (10.5, 43.5) | $3\times{10}^{-17}$ |
| 348.8 | Encephalopathy, not elsewhere classified | 103 | 5 | 20.8 | (8.5, 51.0) | $3\times{10}^{-11}$ |
| 198.5 | Secondary malignancy of brain/spine | 120 | 6 | 20.0 | (8.8, 45.5) | $8\times{10}^{-13}$ |
| 345.1 | Epilepsy | 106 | 6 | 17.9 | (7.9, 40.7) | $6\times{10}^{-12}$ |
| 276.11 | Hyperosmolality and/or hypernatremia | 101 | 6 | 17.1 | (7.5, 38.9) | $1\times{10}^{-11}$ |
| 376.6 | Fluid overload | 98 | 6 | 16.6 | (7.3, 37.9) | $2\times{10}^{-11}$ |
| 979 | Adverse drug events and drug allergies | 179 | 14 | 13.1 | (7.6, 22.6) | $2\times{10}^{-20}$ |
| 290.12 | Dementia with cerebral degenerations | 130 | 10 | 13.1 | (6.9, 25.0) | $4\times{10}^{-15}$ |

**Supplemental Table 5.** Top 10 phenotypes succeeding schizophrenia in men but not in women (total number of schizophrenia patients = 61,453).

| **Phecode** | **Phecode Description** | **Number of Male Schizophrenia Patients** | **Number of Male Non-Schizophrenic Participants** | **Hazard Ratio** | **95% Confidence Interval** | **P-value** |
| --- | --- | --- | --- | --- | --- | --- |
| 038.3 | Bacteremia | 142 | 7 | 20.7 | (9.7, 44.2) | $5\times{10}^{-15}$ |
| 312.3 | Impulse control disorder | 88 | 5 | 18.0 | (7.3, 44.3) | $3\times{10}^{-10}$ |
| 313.3 | Autism | 389 | 24 | 16.6 | (11.0, 25.0) | $1\times{10}^{-40}$ |
| 315.3 | Intellectual Disabilities | 90 | 6 | 15.2 | (6.6, 34.7) | $1\times{10}^{-10}$ |
| 530.2 | Esophageal bleeding | 75 | 6 | 12.9 | (5.6, 29.5) | $2\times{10}^{-9}$ |
| 791 | Gangrene | 53 | 5 | 11.0 | (4.4, 27.6) | $3\times{10}^{-7}$ |
| 264.2 | Failure to thrive | 71 | 7 | 10.3 | (4.7, 22.4) | $4\times{10}^{-9}$ |
| 560.1 | Paralytic ileus | 48 | 5 | 9.9 | (3.9, 24.8) | $1\times{10}^{-6}$ |
| 710.11 | Acute osteomyelitis | 61 | 7 | 9.0 | (4.1, 19.7) | $4\times{10}^{-8}$ |
| 722.9 | Other and unspecified disc disorder | 50 | 7 | 7.3 | (3.3, 16.1) | $8\times{10}^{-7}$ |

**Supplemental Table 6**. Top 10 phenotypes succeeding schizophrenia diagnoses in patients age between 15 and 29 (number of schizophrenia patients in this age group = 13,828).

| **Phecode** | **Phecode Description** | **Number of Schizophrenia Patients** | **Number of Non-Schizophrenic Participants** | **Hazard Ratio** | **95% Confidence Interval** | **P-value** |
| --- | --- | --- | --- | --- | --- | --- |
| 297.1 | Suicidal Ideation | 523 | 3 | 178.5 | (57.4, 555.4) | $3\times{10}^{-19}$ |
| 296.1 | Bipolar | 2,039 | 34 | 110.2 | (71.7, 169.3) | $6\times{10}^{-102}$ |
| 312 | Conduct Disorders | 219 | 3 | 73.1 | (23.4, 228.3) | $2\times{10}^{-13}$ |
| 300.9 | Posttraumatic stress disorder | 404 | 6 | 67.9 | (30.3, 152.1) | $1\times{10}^{-24}$ |
| 316 | Substance addiction and disorders | 1,970 | 36 | 61.5 | (44.3, 85.6) | $2\times{10}^{-132}$ |
| 327.4 | Insomnia | 161 | 3 | 53.8 | (17.2, 168.7) | $8\times{10}^{-12}$ |
| 301.2 | Antisocial/borderline personality disorder | 146 | 3 | 48.5 | (15.5, 152.2) | $3\times{10}^{-11}$ |
| 317.1 | Alcoholism | 270 | 6 | 45.2 | (20.1, 101.5) | $3\times{10}^{-20}$ |
| 296 | Mood disorders | 760 | 18 | 43.9 | (27.5, 70) | $1\times{10}^{-56}$ |
| 305.2 | Eating disorder | 128 | 3 | 42.5 | (13.5, 133.5) | $1\times{10}^{-10}$ |

**Supplemental Table 7**. Top 10 phenotypes succeeding schizophrenia diagnoses in patients age between 30 and 59 (number of schizophrenia patients in this age group = 16,339).

| **Phecode** | **Phecode Description** | **Number of Schizophrenia Patients** | **Number of Non-Schizophrenic Participants** | **Hazard Ratio** | **95% Confidence Interval** | **P-value** |
| --- | --- | --- | --- | --- | --- | --- |
| 317.1 | Alcoholism | 557 | 7 | 82.2 | (39, 172.3) | $4\times{10}^{-31}$ |
| 509.1 | Respiratory failure | 298 | 4 | 76.9 | (28.7, 206.2) | $6\times{10}^{-18}$ |
| 296.1 | Bipolar | 2,618 | 51 | 58.7 | (44.5, 77.5) | 2$\times{10}^{-182}$ |
| 317 | Alcohol-related disorders | 608 | 12 | 52.3 | (29.5, 92.6) | $6\times{10}^{-42}$ |
| 994.2 | Sepsis | 133 | 3 | 45.8 | (14.6, 143.8) | $6\times{10}^{-11}$ |
| 316 | Substance addiction and disorders | 989 | 23 | 45.3 | (30, 68.5) | $5\times{10}^{-73}$ |
| 585.1 | Acute renal failure | 345 | 9 | 39.5 | (20.3, 76.6) | $1\times{10}^{-27}$ |
| 345.3 | Convulsions | 515 | 14 | 37.9 | (22.3, 64.5) | $5\times{10}^{-41}$ |
| 480 | Pneumonia | 320 | 9 | 36.9 | (19, 71.5) | $1\times{10}^{-26}$ |
| 191.11 | Brain cancer | 96 | 3 | 32.2 | (10.2, 101.6) | $3\times{10}^{-44}$ |

**Supplemental Table 8**. Top 10 phenotypes succeeding schizophrenia diagnoses in patients older than 60 years old (number of schizophrenia patients in this age group = 31,286).

| **Phecode** | **Phecode Description** | **Number of Schizophrenia Patients** | **Number of Non-Schizophrenic Participants** | **Hazard Ratio** | **95% Confidence Interval** | **P-value** |
| --- | --- | --- | --- | --- | --- | --- |
| 292.4 | Altered mental status | 3,621 | 53 | 84.2 | (64.2, 110.5) | $4\times{10}^{-225}$ |
| 348 | Other conditions of brain | 1211 | 17 | 75.2 | (46.6, 121.4) | $5\times{10}^{-70}$ |
| 348.1 | Coma; stupor; and brain damage | 810 | 12 | 71.6 | (40.5, 126.7) | $8\times{10}^{-49}$ |
| 290.2 | Delirium due to conditions classified elsewhere | 469 | 7 | 70.9 | (33.6, 150) | $5\times{10}^{-29}$ |
| 296.1 | Bipolar | 1,351 | 377 | 38 | (27.4, 52.7) | $1\times{10}^{-105}$ |
| 292.5 | Transient alteration of awareness | 174 | 5 | 36.9 | (15.2, 89.7) | $2\times{10}^{-15}$ |
| 430.3 | Subdural hemorrhage | 159 | 5 | 32.4 | (13.3, 79.9) | $2\times{10}^{-14}$ |
| 290 | Delirium dementia and amnestic disorders | 1,706 | 59 | 31.3 | (24.1, 40.5) | $6\times{10}^{-149}$ |
| 317.1 | Alcoholism | 325 | 12 | 27.9 | (15.7, 49.6) | $1\times{10}^{-29}$ |
| 819 | Intercranial injury | 132 | 5 | 27 | (11, 66) | $5\times{10}^{-13}$ |

**Supplemental Table 9.** Top 10 significantly enriched phenotypes after the diagnoses of schizoaffective disorders compared with other types of schizophrenia (number of patients with schizoaffective disorder after matching = 3,096).

| **Phecode** | **Phecode Description** | **Number of Patients with Schizoaffective Disorder** | **Number of Patients with Other Schizophrenia** | **Hazard Ratio** | **95% Confidence Interval** | **P-value** |
| --- | --- | --- | --- | --- | --- | --- |
| 216.1 | Screening for malignant neoplasms of the skin | 90 | 7 | 15.8 | (7.3, 34.2) | $3\times{10}^{-12}$ |
| 244.4 | Hypothyroidism NOS | 184 | 17 | 11.5 | (7, 19) | $5\times{10}^{22}$ |
| 338.2 | Chronic pain | 54 | 8 | 7.4 | (3.5, 15.5) | $1\times{10}^{-7}$ |
| 285 | Other anemias | 69 | 11 | 7.3 | (3.7, 14.1) | $5\times{10}^{-9}$ |
| 300.11 | Generalized anxiety disorder | 309 | 96 | 3.6 | (2.9, 4.6) | $7\times{10}^{-28}$ |
| 290.1 | Dementias | 118 | 37 | 3.4 | (2.3, 4.9) | $1\times{10}^{-10}$ |
| 306 | Other mental disorder | 72 | 27 | 3.1 | (2, 4.9) | $5\times{10}^{-7}$ |
| 296.1 | Bipolar | 602 | 245 | 2.9 | (2.5, 3.3) | $6\times{10}^{-43}$ |
| 278.11 | Morbid obesity | 98 | 47 | 2.3 | (1.6, 3.3) | $2\times{10}^{-6}$ |
| 278.1 | Obesity | 96 | 49 | 2.2 | (1.6, 3.1) | $7\times{10}^{-6}$ |

**Supplemental Table 10**. Top 5 significantly depleted phenotypes after the diagnoses of schizoaffective disorder compared with other types of schizophrenia (number of patients with schizoaffective disorder after matching = 3,096).

| **Phecode** | **Phecode Description** | **Number of Patients with Schizoaffective Disorder** | **Number of Patients with Other Schizophrenia** | **Hazard Ratio** | **95% Confidence Interval** | **P-value** |
| --- | --- | --- | --- | --- | --- | --- |
| 1019 | Other causes of morbidity & mortality | 57 | 240 | 0.24 | (0.18, 0.32) | $6\times{10}^{-22}$ |
| 345.3 | Convulsions | 23 | 76 | 0.32 | (0.20, 0.50) | $1\times{10}^{-6}$ |
| 339 | Other headache syndromes | 31 | 89 | 0.37 | (0.25, 0.56) | $2\times{10}^{-6}$ |
| 292.4 | Altered mental status | 39 | 94 | 0.45 | (0.31, 0.65) | $2\times{10}^{-5}$ |
| 296 | Mood disorders | 49 | 111 | 0.45 | (0.32, 0.63) | $4\times{10}^{-6}$ |
